# Changes in intestinal permeability and gut microbiota following diet-induced weight loss in patients with metabolic dysfunction-associated steatohepatitis and liver fibrosis

**DOI:** 10.1080/19490976.2024.2392864

**Published:** 2024-09-28

**Authors:** Dimitrios A. Koutoukidis, Sandi Yen, Paula Gomez Castro, Mariya Misheva, Susan A. Jebb, Paul Aveyard, Jeremy W. Tomlinson, Ferenc E. Mozes, Jeremy F. Cobbold, Jethro S. Johnson, Julian R. Marchesi

**Affiliations:** a Nuffield Department of Primary Care Health Sciences, University of Oxford, Oxford, UK; b NIHR Oxford Biomedical Research Centre, Oxford, UK; c Oxford Centre for Microbiome Studies, Nuffield Department of Orthopaedics, Rheumatology and Musculoskeletal Sciences, University of Oxford, Oxford, UK; d MRC Weatherall Institute of Molecular Medicine, Radcliffe Department of Medicine, University of Oxford, Oxford, UK; e Chemistry Research Laboratory, University of Oxford, Oxford, UK; f NIHR Oxford Health Biomedical Research Centre, Warneford Hospital, Oxford, UK; g Oxford and Thames Valley Applied Research Collaboration, University of Oxford, Oxford, UK; h Oxford Centre for Diabetes, Endocrinology and Metabolism, Radcliffe Department of Medicine, University of Oxford, Oxford, UK; i Oxford Centre for Clinical Magnetic Resonance Research, Radcliffe Department of Medicine, University of Oxford, Oxford, UK; j Department of Gastroenterology and Hepatology, John Radcliffe Hospital, Oxford University Hospitals NHS Foundation Trust, Oxford, UK; k Division of Digestive Diseases, Department of Metabolism, Digestion and Reproduction, St Mary’s Hospital, Imperial College London, London, UK

**Keywords:** Diet, weight loss, intestinal permeability, gut microbiota, metabolic dysfunction-associated liver disease

## Abstract

Weight loss improves metabolic dysfunction-associated steatohepatitis (MASH). We investigated whether there were associated changes in intestinal permeability, short-chain fatty acids (SCFAs), and gut microbiota, which are implicated in the pathophysiology of MASH. Sixteen adults with MASH, moderate fibrosis, and obesity received a low-energy total diet replacement program for 12 weeks and stepped food re-introduction over the following 12 weeks (ISRCTN12900952). Intestinal permeability, fecal SCFAs, and fecal microbiota were assessed at 0, 12, and 24 weeks. Data were analyzed using mixed-effects linear regression and sparse partial least-squares regression. Fourteen participants completed the trial, lost 15% (95% CI: 11.2–18.6%) of their weight, and 93% had clinically relevant reductions in liver disease severity markers. Serum zonulin concentrations were reduced at both 12 and 24 weeks (152.0 ng/ml, 95% CI: 88.0–217.4, *p* < 0.001). Each percentage point of weight loss was associated with a 13.2 ng/mL (95% CI: 3.8–22.5, *p* < 0.001) reduction in zonulin. For every 10 ng/mL reduction in zonulin, there was a 6.8% (95% CI: 3.5%-10.2, *p* < 0.001) reduction in liver fat. There were reductions in SCFA and alpha diversity evenness as well as increases in beta diversity of the gut microbiota at 12 weeks, but the changes did not persist at 24 weeks. In conclusion, substantial dietary energy restriction is associated with significant improvement in MASH markers alongside reduction in intestinal permeability. Changes in gut microbiota and SCFA were not maintained with sustained reductions in weight and liver fat, suggesting that microbiome modulation may not explain the relationship between weight loss and improvements in MASH.

## Introduction

Metabolic dysfunction-associated steatohepatitis (MASH), previously known as nonalcoholic steatohepatitis (NASH), with liver fibrosis is an advanced form of metabolic dysfunction-associated steatotic liver disease (MASLD) associated with obesity. It is estimated to affect approximately 5% of the adult population worldwide and is associated with an increased risk of liver and cardiovascular events.^1–3^ No pharmacological treatment has been approved for MASH, and advice to lose weight remains the mainstay of treatment.^4^

A minimum of 7–10% weight loss is recommended to improve MASH in people with obesity, with greater weight loss leading to greater benefits.^5–7^ We have previously shown that a low-energy total diet replacement program led to an average 15% weight loss at 24 weeks, demonstrated a favorable safety profile, and improved markers of liver disease severity and cardiometabolic markers among people with MASH and obesity.^8^ However, the mechanism by which weight loss exerts its effects on the liver remains incompletely understood.

The multiple parallel hits hypothesis points to the microbiome and the intestinal barrier dysfunction as key drivers in the pathophysiology of MASH.^9^ Barrier dysfunction may be mediated by changes in the gut microbiome through multiple mechanisms. For example, a reduced capacity to synthesize short-chain fatty acids (SCFAs), such as butyrate, has been shown to increase tight junction formation.^10^ Barrier dysfunction also permits the translocation of bacteria and bacterial products (i.e. endotoxins and metabolites) which may directly contribute to the development of MASH.^11^

In humans, retrospective and cross-sectional data show that people with MASLD and MASH are significantly more likely to have high intestinal permeability than healthy controls (OR = 5 and 7, respectively).^12,13^ MASH and fibrosis are also cross-sectionally associated with a decrease in the alpha diversity (i.e., a combination of the number of taxa and the distribution of abundances of the taxa) of the gut microbiota composition compared with both healthy controls and people with obesity, but not MASH.^14,15^ Preclinical studies support a causal role for the gut microbiota in MASH and suggest that large weight loss reduces markers of intestinal permeability.^14,16^ Large weight loss in the general population with obesity is also associated with both increases in the alpha diversity of the gut microbiota and reductions in intestinal permeability.^17^ Therefore, it is plausible that substantial weight loss through dietary energy restriction might improve disease severity by modulating intestinal permeability, SCFAs, and the gut microbiota in people with MASH.

There remains a paucity of studies exploring the associations between weight loss and changes in intestinal permeability, microbiome composition, and function in humans. Empirical data are needed to evaluate the role of the human gut microbiome in weight loss-associated MASH treatment. In this context, our aim was to quantify intestinal permeability, SCFAs, and gut microbiota following a dietary weight loss intervention in patients with MASH and liver fibrosis.^8^ In doing so, we sought to evaluate whether weight-loss associated improvements in markers of liver disease severity are linked to increases in alpha diversity, SCFA production, and reductions in intestinal permeability.

## Methods

### Participants and intervention

This analysis was based on a previously reported, prospectively registered weight loss trial (ISRCTN12900952).^8,18^ All research was conducted in accordance with both the Declarations of Helsinki and Istanbul, all research was approved by the appropriate ethics review committees (19/LO/1856, 16/YH/0247) and written consent was given by all participants. In brief, participants with a body mass index ≥30 kg/m^2^, stable weight, MASH, histologically proven stage 2–3 fibrosis, and no evidence of other liver diseases were enrolled in a single-arm trial. Participants were asked to follow a 24-week intervention that received behavioral support from a registered dietitian (Oviva, United Kingdom). During the first 12 weeks, participants replaced all foods with nutritionally replete meal replacement products, providing approximately 880 kcal/day with ~80 g protein/day and ~14–20 g soluble fiber/day (Optifast, Nestle Health Science, UK). In the next 12 weeks, participants gradually reduced their meal replacement products and reintroduced food-based meals in line with healthy eating guidelines. During weeks 13–14, participants were supported to consume 3 products a day and one low-carbohydrate meal based on protein and non-starchy vegetables. During weeks 15–16, the number of products reduced to 2 and an additional meal based on the T-plate model (1/2 plate non-starchy vegetables, ¼ plate carbohydrate, and ¼ plate protein) was added. During weeks 17–23, participants were supported to have 1 meal replacement product a day in addition to real food tailored to their individual preferences. They transitioned to only food on week 24. Throughout the last 12 weeks, participants remained on a reduced energy diet with the amount of energy tailored to whether individuals wanted to maintain their lost weight or lose more weight. If they regained >2 kg, they were offered to return up to 4 weeks to total diet replacement.

### Body composition and liver fat

Weight was measured and fat mass was estimated using bioelectrical impedance (TANITA SC-240 MA, Tanita). The participants fasted for at least 4 hours before the scans. Magnetic resonance imaging was performed by trained operators using a Siemens Prisma 3T scanner (Siemens Healthineers, Erlangen, Germany). MRI proton density fat fraction (PDFF) was determined using a multiple-echo gradient-recalled echo sequence. The details are provided in Supplementary Section 1. Additionally, we measured liver stiffness based on vibration-controlled transient elastography and magnetic resonance elastography, as well as liver biochemistry and controlled attenuation parameters, as described previously.^8^

### Measuring markers of intestinal permeability

Zonulin, a regulator of gut epithelial and endothelial tight junctions, is a marker of intestinal permeability and has been proposed as a noninvasive marker of MASH disease progression.^19,20^ Zonulin modulates reversible tight junction disassembly and dysregulation of this pathway leads to increased mucosal permeability. In addition to zonulin, our primary measure, we measured serum lipopolysaccharide-binding protein (LBP), a marker of endotoxemia and translocation of lipopolysaccharide across the intestinal barrier.^21^ Zonulin and LBP levels are elevated in MASLD/MASH and seem to be responsive to weight loss in non-MASLD cohorts.^17,22,23^

Serum samples were stored at −80°C until analysis. All measurements were assessed using commercially available ELISA kits [Zonulin: Elabscience, E-EL-H5560 (detection range 0.78–50 ng/mL, sensitivity 0.47 ng/mL) and LBP: Hycult Biotech, HK315–01 (detection range 4.4 to 50 ng/ml, sensitivity 4.4 ng/ml)]. Both intra- and inter-assay coefficients of variation were <10%. Samples were tested in triplicates in 96 well plates at the Oxford Centre for Microbiome Studies following the manufacturer’s instructions.

### Gut microbiota sampling and DNA extraction

The study participants received a sample collection kit with instructions (Supplementary Section 2) and a collection tube with >99% ethanol for fixed fecal sample collection and storage. The samples were produced at home following each study visit at 0, 12, and 24 weeks and posted with next-day delivery to the University of Oxford, where the samples were stored at −80°C until analysis. They all reported no antibiotic use in the week preceding sample collection.

Fecal samples were extracted manually using the QIAamp PowerFecal Pro DNA kit (Qiagen 51,804) according to the manufacturer’s instructions. Sample homogenization in PowerBead tubes was performed using a Qiagen Vortex adapter coupled to a Vortex Genie 2 for 20 min at the maximum speed. Initial DNA quantification was performed on a NanoDrop (Thermo Scientific) and DNA concentration was adjusted to within the desired mass range for sequencing and plated on a 96 well, low profile, skirted PCR plate (4titude) for sequencing submission. A microbial community DNA standard (D6305; ZymoBIOMICS) was included as a sequencing positive control, and an extraction blank (PBS) was used as a negative control.

### Targeted mass spectrometry analysis of fecal short-chain fatty acids

The ethanol-fixed fecal samples were processed and normalized for SCFA-targeted metabolomics. Prior to any subsampling (for microbiota or SCFA measurements), wet and dry sample weights were calculated (Supplementary Section 4). Samples were homogenized manually by adding sterilized 7 mm steel bead into each tube and vortexing at full speed for 5–10 min. The samples were centrifuged at 3,000 × g for 10 min at 4°C. The supernatants were transferred to a 0.45 µm PTFE spin column (Sigma-Aldrich, UFC30LH25) and centrifuged at 12,000 × g at 4°C for 5 min. Filtered samples were transferred into vials for further downstream filtering with 10 MWCO Amicon filters and for targeted SCFA analysis using ion-chromatography mass spectrometry (IC-MS).

The targeted SCFA IC-MS method, detailed in Supplementary Section 5, was designed to specifically identify SCFAs and their isomers. Acetic, butyric, isobutyric, isovaleric, propionic, and valeric acids were analyzed. A standard curve was generated for each SCFA and used for sample calculations. All peaks for SCFAs were integrated into the standard curve and the samples, and the peak area was used to calculate the concentration of SCFAs in the samples.

SCFA concentrations were adjusted according to stool wet weight (SCFA concentration divided by sample wet weight, as measured before subsampling for dry weight calculations). Wet-weight adjustment was indicated to be more pertinent than dry-weight adjustment, as it eliminated variability in stool consistency inherent to major changes in diet (Supplementary Section 4). SFCA levels were reported as mM/mg of stool. Changes in SCFA profiles throughout the study were assessed using a restricted permutation PERMANOVA. The variability observed within each time point was assessed by dispersion, as measured by the Euclidean distance to the group (time point) centroid.

### 16S rRNA gene amplicon sequencing and processing

For bacterial 16S rRNA gene amplicon sequencing, the V3 and V4 regions of the 16S rRNA gene were amplified from the genomic DNA, as detailed in Supplementary Section 3. Briefly, Sequences were obtained using the Illumina MiSeq v3 flow cell as 2 × 300 bp paired-end reads. Raw sequence quality was assessed using FastQC (v0.11.07) and subsequently processed using DADA2 implemented by OCMS_16S (https://github.com/OxfordCMS/OCMS_16S).^24,25^ Taxonomy was assigned to amplicon sequence variants (ASVs) using DADA2 against NCBI RefSeq (Supplementary Section 3). Four samples were excluded from the analysis because they had an insufficient read depth (<9000 reads), as indicated by the richness rarefaction curve. Following quality control, the median read depth was 289,938 reads/sample.

### Gut microbiota diversity

We assessed the alpha diversity of the gut microbiota using Shannon’s D, which combines the observed number of taxa (richness) and the observed distribution of their respective abundance (evenness). A higher value indicates more taxa and even higher abundance. Statistical significance of alpha diversity was assessed using a mixed-effects linear model (see the Statistics section for details). We assessed beta diversity using the Bray-Curtis distance, which assesses the pairwise dissimilarity in microbial abundances of taxa between samples. A value of 0 indicates that samples share an identical microbiota composition, and a value of 1 indicates no overlap in the observed taxa. The statistical significance of differences in microbiota composition between samples (i.e., beta diversity) throughout the study was assessed using a restricted permutational multivariate analysis of variance (PERMANOVA) based on the Bray-Curtis distance. Beta diversity analysis is presented at the phylum level; however, analyses at all other taxonomic levels are available in the supplement.

## Data handling

Data processing and normalization were performed on a case-by-case basis. Data processing steps are detailed in Supplementary Section 6.

## Statistics

We conducted our analysis of available cases at each time point using an intention-to-treat approach (i.e., regardless of adherence to the intervention). To examine the change in each variable throughout the study, we ran mixed-effects linear models (MELMs) with the variable of interest as the dependent variable, the participant as a random effect, and the timepoint as a fixed effect. If the time point factor was statistically significant (*p* < 0.05), we assessed whether changes in the variable of interest were associated with percentage weight change throughout the study (0–12 and 12–24 weeks). Each of these latter MELMs included the participant as a random effect and a multiplicative interaction between time point and percentage weight change to assess whether the relationship differed between 0–12 and 12–24 weeks. All significant MELMs were followed up with a *post-hoc* test to measure pairwise differences in LS means for all factors in the model. This analytical approach was applied to zonulin, SCFA, alpha diversity, beta diversity, and dispersion measures. All MELM terms and post-hoc test results are presented in the Supplement.

We replicated the above analysis with change in microbiota alpha diversity (Shannon’s D index and evenness) as independent variables and changes in liver severity markers as dependent variables. Variables were assessed in a pairwise manner, so a total of 16 (8 liver severity markers X 2 alpha diversity measures) MELMs were performed. The relationship between change in zonulin (dependent) and change in each SCFA (independent) was assessed in a similar manner.

Analyses were conducted in R version 3.1.2, using the lme4, lmerTest, mixOmics, OCMSutility, ALDEx2, vegan, and rstatix packages.^26^

## Results

**Figure 1.**
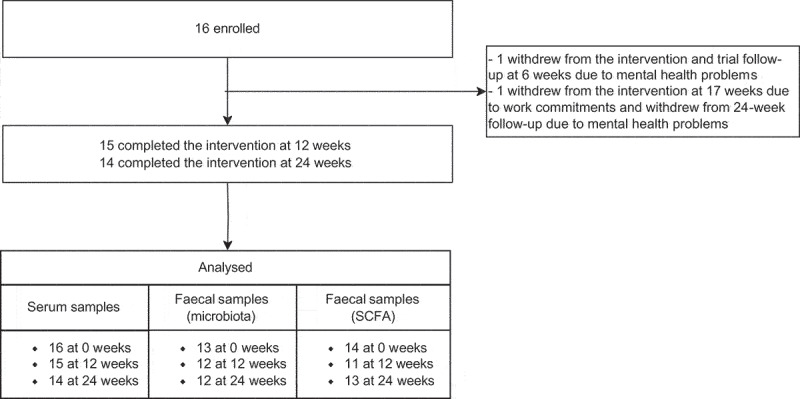
Participant and sample flow.

All 16 participants provided weight and blood samples at baseline, 15 at 12 weeks, and 14 at 24 weeks (Figure 1). Weight was significantly reduced at 12 weeks (−14.5% (95% CI: −17.6 to −11.8) and remained stable between 12 and 24 weeks [−0.3% (95% CI: −2.80 to 2.85)]. Changes in liver disease indices among these participants have been previously reported.^8^ Following quality control, there were acceptable fecal samples for microbiota analysis for *n* = 13 participants at baseline and *n* = 12 thereafter, as well as for *n* = 14, *n* = 11, and *n* = 13 for SCFA analysis (Figure 1). The participant characteristics are shown in Table 1.

**Table 1.** Demographic characteristics of those enrolled, those with serum samples for the intestinal permeability analysis at 24 weeks and those with missing samples at 24 weeks.

	Enrolled	Valid for IP analysis at 24 weeks	Valid for microbiota analysis at 24 weeks
Characteristic	n = 16	n = 14	n = 12
Age, median (IQR)	58 (38-62)	59 (51-62)	59 (55-61)
Sex, female	5 (31.2)	4 (28.6)	4 (33.3)
Ethnicity			
Asian	1 (6.2)	1 (7.1)	0 (0)
White	15 (93.8)	13 (92.9)	12 (100)
Annual household income			
<£15.5k	3 (18.8)	2 (14.3)	2 (16.7)
£15.5-25k	2 (12.5)	2 (14.3)	2 (16.7)
£26-39k	6 (37.5)	6 (42.9)	4 (33.3)
≥£40k	5 (31.2)	4 (28.6)	4 (33.3)
Smoking status			
Never	8 (50)	6 (42.9)	4 (33.3)
Previous	6 (37.5)	6 (42.9)	6 (50)
Current	2 (12.5)	2 (14.3)	2 (16.7)
At least one medication for type 2 diabetes	6 (37.5)	5 (35.7)	5 (41.7)
At least one medication for hypertension	7 (43.8)	6 (42.9)	5 (41.7)
Alcohol units in the past 7 days, median (IQR)	0 (0-3)	0 (0-3)	0 (0-3)
Baseline BMI, kg/m^2^, median (IQR)	36.2 (34.3, 39.1)	35.6 (32.2, 38.4)	35.6 (34.3, 38.0)
Baseline weight, kg, median (IQR)	116.6 (93.2, 123.4)	112.0 (89.1, 119.9)	112.0 (93.2, 118.9)
Weight loss at 24 weeks from baseline, %, median (95% CI)	−15.0 (−18.6 to −11.2)	−15.0 (−18.6 to −11.2)	−14.9 (−19.2 to −10.5)
Baseline liver fat (PDFF), %, median (IQR)	18.6 (13.3, 21.9)	18.6 (12.4, 21.8)	18.6 (13.3, 21.9)
PDFF change at 24 weeks from baseline, % points, median (95% CI)	−13.1 (−16.7 to −8.9)	−13.1 (−16.7 to −8.9)	−12.9 (−17.9 to −8.2)

Data are n (%) unless otherwise specified. PDFF: Proton density fat fraction, IP: intestinal permeability

## Intestinal permeability

**Figure 2.**
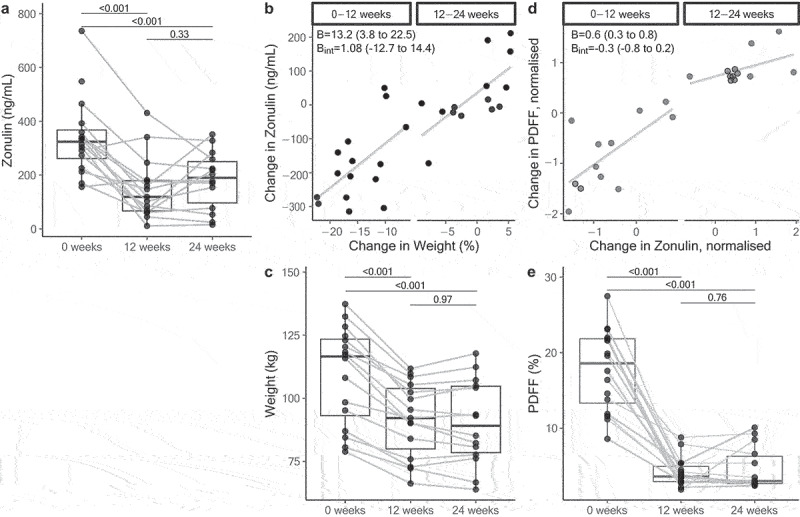
(a) Median (IQR) of zonulin at each time point and changes for each individual participant. (b) Association between percentage weight change and changes in zonulin at 0-12 weeks and 12-24 weeks. (c) Median (IQR) of weight at each timepoint. (d) Association between change in liver fat (PDFF, normalized) and change in zonulin (normalized) at 0-12 weeks and 12-24 weeks. (e) Median (IQR) of liver fat (PDFF) at each timepoint. In panels B and D, beta is the coefficient from the mixed model and b_int_ is the coefficient of the interaction term with their corresponding 95% confidence intervals.

The baseline and absolute changes in zonulin concentrations at each time point are shown in Figure 2(a). There was a significant reduction in zonulin at 12 weeks (−184.7 ng/ml, 95% CI: −248.5, −122.2, p_adj_ <0.001) which remained significantly lower than baseline at 24 weeks (−152.0 ng/ml, 95% CI: −217.4, −88.0, *p* < 0.001). Each percentage point of weight loss was associated with a 13.2 ng/mL (95% CI: 3.8, 22.5, p_adj_ <0.001) reduction in zonulin for the entire duration of the study. This relationship did not differ between the periods of acute weight loss (0–12 weeks) and weight stability (12–24 weeks) (Figure 2(b) and 2(c)). There was no significant change in LBP (Figure S1); therefore, it was not included in any subsequent univariate MELM analyses.

Exploring all liver outcomes against changes in intestinal permeability markers in a multivariate model revealed a positive relationship between reductions in zonulin levels and reductions in PDFF (0.60, 95% CI: 0.31 to 0.88, Figure 2(d) and 2(e)). For every 10 ng/ml change in zonulin, there was a 6.8% (95% CI: 3.5%, 10.2, *p* < 0.001) reduction in PDFF with no significant interaction between the two dietary periods (−0.30, 95% CI: −0.8 to 0.21), indicating that there was no evidence that the relationship between change in PDFF and change in zonulin was statistically different between the acute weight loss and weight stability periods.

## Short-chain fatty acids

**Figure 3.**
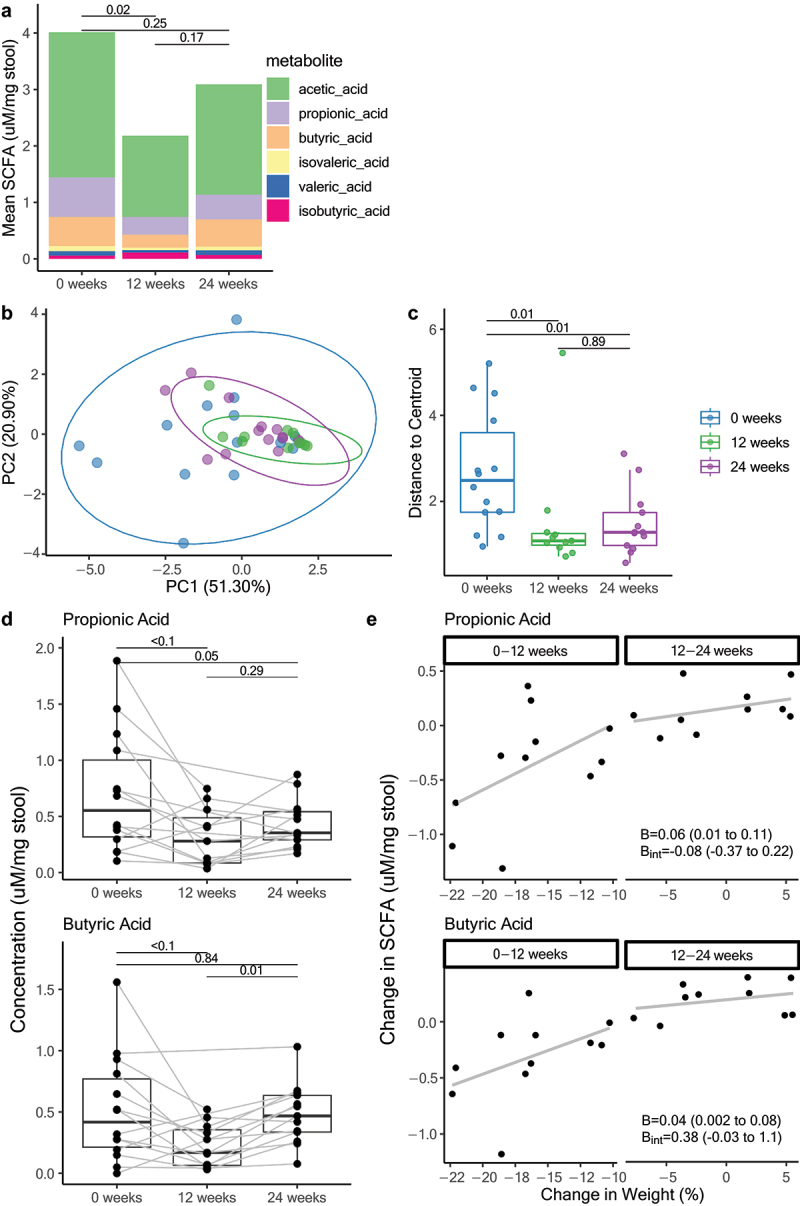
(a) mean concentration of targeted fecal short chain fatty acid adjusted for stool weight at each time point. (b) PCoA of SCFA profiles for each individual at each timepoint. Baseline in blue, 12 weeks in green, 24 weeks in purple. (c) Dispersion of SCFAs as measured by Euclidean distance of each sample to the group centroid at each time point. (d) fecal propionic acid at each timepoint. (e) Faissal butyric acid at each timepoint. (f) Change in propionic and butyric acid associated with change in weight at 0 to 12 weeks and 12 to 24 weeks. Beta is the coefficient from the mixed model and b_int_ is the coefficient of the interaction term with their corresponding 95% confidence intervals.

The cumulative fecal SCFA concentrations of the targeted SCFA (acetic acid, butyric acid, isobutyric acid, isovaleric acid, propionic acid, and valeric acid) are shown in Figure 3(a). Compared with baseline (4.0 uM/mg stool, 95% CI: 2.9 to 5.1), the cumulative SCFA concentration was significantly reduced (−1.8, 95% CI: −3.1 to −0.4) after the acute weight loss phase, but was not different from baseline at 24 weeks (−0.8, 95% CI −2.1 to 0.5). PCoA in Figure 3(b) showed that participant SCFA profile heterogeneity is explained by timepoint (PERMANOVA R^2^ = 0.13, *p* < 0.01), where the inter-individual variation was the largest at baseline (2.7, 95% CI: 2.1 to 3.3), and converged to become more similar at 12 weeks (−1.2, 95% CI: −2.0 to −3.5). Participants’ SCFA profiles maintained their inter-individual similarity across the 12–24-week weight stability period (−1.1, 95% CI: −2.0 to −0.4), as illustrated by the dispersion of participants from the group centroid at each time point (Figure 3(c)).

Exploring individual SCFAs, propionic acid (baseline: 2.6, 95% CI: 1.8 to 3.3) was significantly reduced at 12 weeks (−0.4, 95% CI: −0.6 to −0.1) and remained lower than the baseline at 24 weeks (−0.2, 95% CI: −0.5, −0.005) (Figure 3(d)). Butyric acid levels were significantly reduced at 12 weeks (−0.3, 95% CI: −0.5 to −0.08) but returned to levels no different from baseline at 24 weeks (−0.02, 95% CI: −0.2 to 0.2) (Figure 3(d)). Acetic, isobutyric, valeric, and isovaleric acids did not change significantly. There were indications of associations between change in weight and change in butyric (*p* = 0.09) or propionic (*p* = 0.06) acid levels, but these did not reach statistical significance (Figure 3(e)). Changes in individual SCFAs were not associated with changes in zonulin or changes in any of the liver markers.

## Fecal microbiota diversity

**Figure 4.**
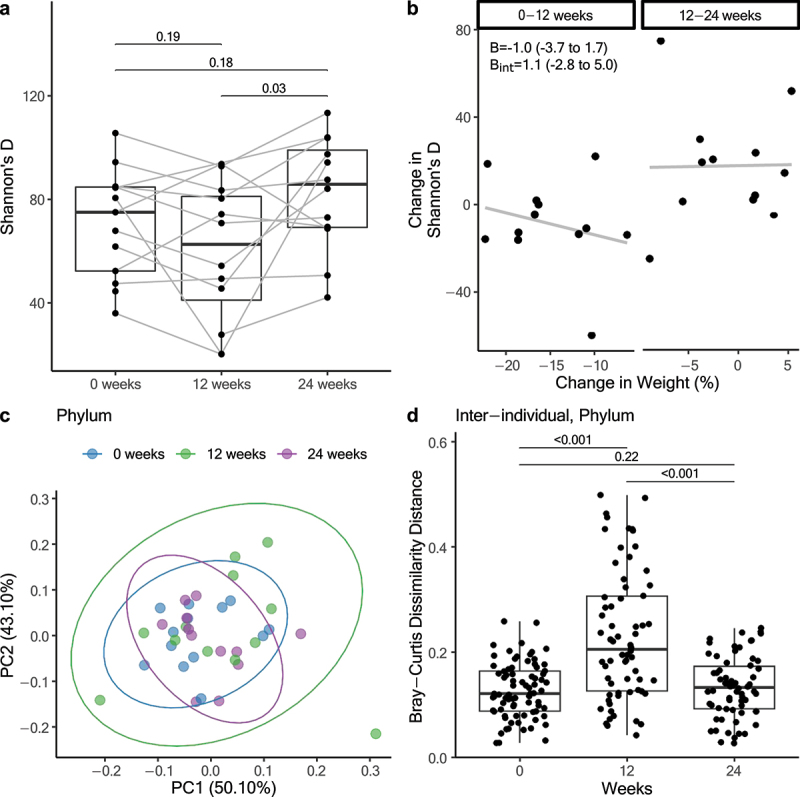
(a) Within-sample alpha diversity measured by Shannon’s D. (b) Association between change in Shannon’s D and percentage weight change between 0 to 12 weeks and 12 to 24 weeks. (c) PCoA of phylum level microbiota (bray-curtis distance of relative abundances). Samples from 0 weeks in blue, 12 weeks in green, 24 weeks in purple. Beta is the coefficient from the mixed model and b_int_ is the coefficient of the interaction term with their corresponding 95% confidence intervals. (d) Inter-individual comparison of the abundance of taxa at phylum level between samples. Each individual is compared against all other individuals at each timepoints.

Shannon’s D index (Figure 4(a)) was significantly different between the three time points (*p* = 0.009) with *post-hoc* tests indicating that this was due to a significant increase in alpha diversity between 12 and 24 weeks, but no significant difference between baseline and 12 or 24 weeks. Exploring the components of Shannon’s D index indicated that the number of taxa (richness) was not significantly different between time points. However, the distribution of taxon abundance (evenness) decreased between baseline and 12 weeks, increased significantly between 12 and 24 weeks, and showed no significant difference between baseline and 24 weeks. Changes in alpha diversity were not associated with changes in weight (Figures 4(b) and S2) or changes in liver outcomes (Figure S2, Table S7).

Beta diversity assessments indicated that the composition of the gut microbiome did not change significantly between baseline, 12 weeks, and 24 weeks, as indicated by PERMANOVA (R2 ≤ 0.05, p_adj_ > 0.05 for all taxonomic levels, Figure S3). However, it was notable that dispersion (i.e., inter-individual variability in gut microbiome composition) was greater at 12 weeks (Figure 4(c)), as reflected by a statistically significant increase in pairwise Bray-Curtis distances at this time point compared to baseline and 24 weeks (Figure 4(d)). These trends in microbiome composition were consistent when summarizing microbiome data at different taxonomic levels, with statistical significance achieved at the class, order, and family levels (Figure S3). Figure S4 shows the average relative abundance of the most abundant taxa at each taxonomic level and Figure S5 shows the taxa that were differentially abundant between baseline and 12 weeks.

## Discussion

Zonulin, our primary marker of intestinal permeability, decreased in a dose-dependent manner along with weight loss in patients with MASH and liver fibrosis. This reduction in zonulin also exhibited a dose–response relationship with reduction in liver fat. Cumulative SCFA were reduced at 12 weeks, driven by reductions in propionate and butyrate levels. However, by week 24, cumulative and individual SCFA levels, except propionate, were comparable to those at baseline. The composition evenness of the microbiota, but not richness, was reduced following acute weight loss, whereas the beta diversity showed the opposite pattern. Both measures of diversity returned to levels that were not different from baseline after the weight stability phase.

These data provide mechanistic insights into the reduction in intestinal permeability with weight loss and improvement in MASH disease severity. The observed changes in zonulin are also consistent with data from a systematic review and meta-analysis showing that intestinal permeability markers reduce in a dose–response manner in people with obesity in response to weight loss.^17^ The amount of liver fat resolved to normal levels in 71% of the participants, and the reduction in zonulin exhibited a dose–response relationship with the reduction in liver fat. This finding is consistent with evidence from a systematic review of lower intestinal permeability in patients with MASLD compared to healthy controls.^27^ From a pathophysiological perspective, reduced intestinal permeability could be mechanistically linked to improved systemic inflammation and immune function, as a disrupted immune function is a key pathway in advanced liver disease.^28^

Higher alpha diversity is typically correlated with beneficial health states,^29,30^ and lower alpha-diversity is associated with MASLD.^15^ However, we observed no evidence of change in overall alpha diversity, which comprised a temporary expansion of a few highly abundant bacteria (i.e., less evenness), but no loss or gain of certain taxa. Concurrently, the composition became more variable after acute weight loss. However, despite sustained reductions in weight and liver fat after the weight stability phase, no changes in alpha and beta diversity were maintained, with both returning to levels no different from baseline. Although changing the dietary composition (e.g., increasing fiber intake) can modulate microbiota composition,^31^ changes in microbiota composition did not seem to play an essential role in reducing liver fat in the context of a tightly controlled, nutritionally balanced, energy-restricted diet. Therefore, although gut microbiota differences are linked to the development of MASLD, our study demonstrated that significant improvements in MASH disease severity markers can be achieved with substantial energy restriction in the absence of lasting alterations in microbiota composition. Thus, we found no evidence to support the therapeutic value of changing microbiota to improve MASH in this context.

Contrary to our expectations, we observed a decrease in cumulative SCFA levels in response to acute weight loss. While SCFA, such as propionate and butyrate, has been shown to contribute to reductions in intestinal permeability and liver fat,^32–35^ fecal SCFA did not appear to be the driver of the observed improvements in intestinal permeability or relate to changes in weight or liver fat in our study. It is possible that the benefits of SCFA on intestinal permeability are localized to more proximal regions of the gastrointestinal tract and are not well captured in the stool, or that intestinal permeability with weight loss in MASH changes through pathways that do not substantially involve SCFA.^36^ This suggestion is consistent with our previous systematic review of weight loss interventions that showed no evidence of change in SCFA after weight loss interventions.^17^ Indeed, bile acids, tryptophan indoles, microbial lipids (ethanolamine degradation), and protein metabolism (phenylacetic acid production) have been shown to contribute to intestinal permeability in obesity and steatosis settings^37–39^ and should be explored in future studies.

Strengths of the study include the longitudinal design, the tightly controlled dietary intervention, substantial reductions in weight and markers of liver disease severity, and use of standardized protocols. To our knowledge, this is the first study to examine the concurrent functional responses of both the participants/hosts and microbiota, thus providing a comprehensive characterization of the effects of substantial energy restriction in the context of MASH in human cohorts. This study contributes to our understanding of the role of the gut-liver axis in MASH severity.

Limitations of this secondary analysis include the small sample size, reducing power to detect associations, and lack of a control group that precludes causal inferences. Given the lack of a reference measure for intestinal permeability, the challenges of conducting the sugar absorption test, and the ethanol preservation of fecal samples, we opted to use a combination of serum markers to assess intestinal permeability. However, serum zonulin level has an acceptable correlation with urinary sugar excretion.^40^ Future studies should also consider, where feasible, repeat biopsies and tissue staining to assess changes in epithelial integrity. The standardization and lack of variety in the diet over the first 12 weeks allowed us to perform a tightly controlled intervention in the real world, but might have contributed to the observed initial reduction in alpha diversity. Equally, the increase in the variety of the diet during the food re-introduction in the weight stability phase might have contributed to the subsequent increase in diversity. The large variability between individuals and the small sample size hampered our ability to provide precise evidence.

In summary, substantial dietary energy restriction was associated with significant improvement in MASH markers alongside reduction in intestinal permeability. Changes in gut microbiota and SCFA were not maintained despite sustained reductions in weight and liver fat, suggesting that microbiome modulation may not explain the relationship between weight loss and improvements in MASH.

## Supplementary Material

Supplemental Material

## List of abbreviations

LBP: Lipopolysaccharide-binding protein
MASH: metabolic dysfunction-associated steatohepatitis
MASLD: Metabolic dysfunction-associated steatotic liver disease
SCFAs: Short-chain fatty acids

## Clinical trial registration

ISRCTN12900952, prospectively registered on 14/02/2020

## Data sharing

Data from the bacterial 16S rRNA gene amplicon sequencing were submitted to Annotare (accession number: MTAB-14061) with additional metabolomics and biomarkers data submitted to MetaboLights (MTBLS10316). Deidentified individual participant data are available from the corresponding author upon request. All proposals requesting data access will need to complete a data request form with details of the research question and the analysis plan.
