# Supplementary material for: Changes in intestinal permeability and gut microbiota following diet-induced weight loss in patients with metabolic dysfunction-associated steatohepatitis and liver fibrosis
Source: Gut Microbes. 2024 Sep 28;16(1):2392864. doi: 10.1080/19490976.2024.2392864 (PMC11444513; doi:10.1080/19490976.2024.2392864)
Supplement: Supplemental Material [file KGMI_A_2392864_SM2240.zip › Supplement.pdf]

Supplement – Koutoukidis et al.

## 1. Measurements

Height was measured at baseline barefoot on a stadiometer.

MRI PDFF was determined using a multiple-echo gradient-recalled echo sequence with 12 echo times, $TR/TE_{min}$ = 15/1.1 ms, $\Delta TE$ = 1.1 ms, slice thickness 10 mm, and FA = 3°, bandwidth 1560 Hz/px. PDFF maps were reconstructed using the IDEAL algorithm.[1]

A standard shortened modified Look-Locker (shMOLLI) sequence was used to acquire T1 maps [2]. Three inversion times were followed by 5, 1 and 1 single shot bSSFP images (TR/TE=2.43/1.05 ms, $TI_{min}$=100, 1100, 2100, 3100, 4100, 180, 260 ms) with FOV = 440 mm´ 330 mm, slice thickness 8 mm, FA = 35°, and BW = 965 Hz/px. Iron correction was performed by Perspectum to yield cT1 values [3, 4].

MRE was acquired using a gradient echo sequence with the following parameter: TR/TE=50/16 ms, encoding frequency = 60 Hz, n=4 slices, FOV = 400 mm ´ 300 mm, slice thickness= 5 mm, FA = 25°, BW = 260 Hz/px. Liver stiffness measurements were averaged across the four slices as recommended by the Quantitative Imaging Biomarkers Alliance [5].

The VCTE scan (FibroScan®, Echosens, France) was performed on the right liver lobe and the probe (M or XL) was selected based on the manufacturer's automatic probe selection tool. The scan provides a measurement of liver stiffness in kilopascals (kPa), an average estimate of the velocity of the tissue at a shear wave produced by the vibration generated by the probe at a frequency of 50 Hz which reflects liver fibrosis. It also provides a measurement of the controlled attenuation parameter (CAP) in dB/m, an average estimate of ultrasound attenuation at 3.5 MHz that is indicative of liver fat. At least 10 valid individual measurements with an interquartile range/med<30% were required to consider the scan valid

## 2. Instructions for faecal sample collection

*Patient Instructions for stool sample collection (Air Sea postal kit) - University of Oxford*
*[Version 1.0]*

UNIVERSITY OF OXFORD

Oxford Centre for Microbiome Studies

### Instructions to patients for collection of stool samples

You will have been given the following stool collection kit:

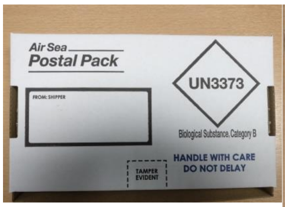


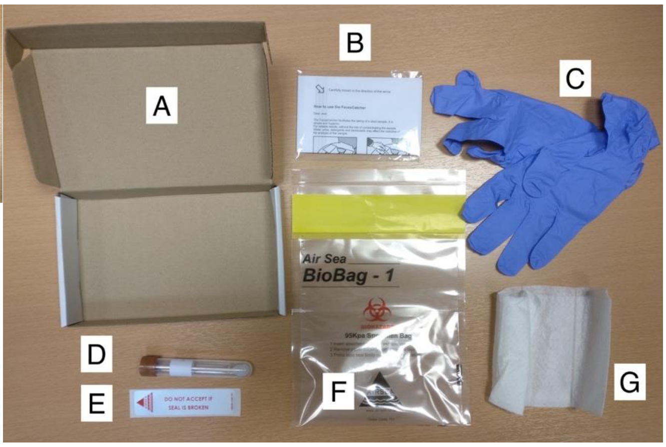


A. Air Sea postal box (including instructions and questionnaire)
B. Stool catcher
C. Gloves
D. Clear sample bottle
E. Silver tamper seal
F. BioBag
G. Sample protector

**COLLECTING THE STOOL SAMPLE**

1. Remove the stool catcher (item B) from its packaging. Pull the sides of the stool catcher apart from each other in the direction of the arrows on the catcher. Using the sticky edges, place on toilet bowl (ensure rim of bowl is dry) as shown below on the photos.

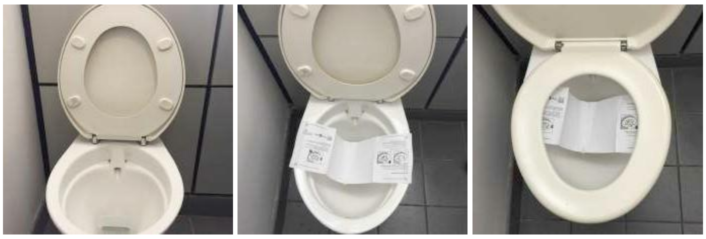

*Step 1* *Step 2* *Step 3*

2. After stool is deposited onto the stool catcher, put on the gloves (item C).

3. Take the clear sample container (item D), containing a small amount of liquid preservative. Use the lid with scoop from this container to place only one scoop of stool into the clear container. Make sure that you hold this bottle up-right so that the liquid does not spill

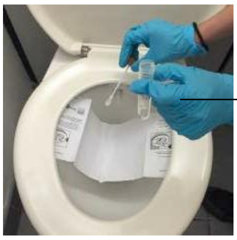

*Collecting sample into clear bottle*

Hold clear bottle upright so as not to tip out liquid.

.

**HOW TO PACKAGE THE SAMPLE CONTAINER**

4. Wrap the clear sample container (item D) in the sample protector (item G), then place in the BioBag (item F). Seal the bag by removing the protective tape from the yellow strip and pressing down.

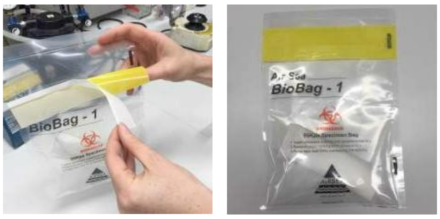


*Place bottle wrapped in tissue packet into the BioBag.*

5. Place the BioBag in the Air Sea postal box (item A)

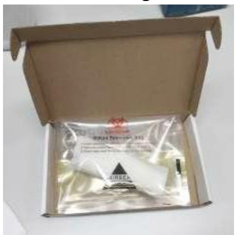

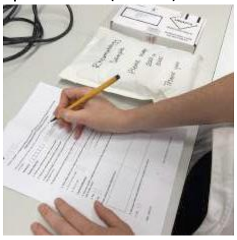

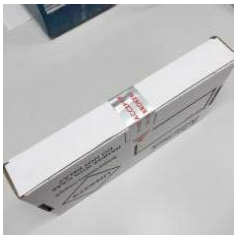

6. Now place the completed questionnaire into the same box. We appreciate you taking the time to fill out this questionnaire as it is a necessary part of our study.
7. Close the postal box and place the silver seal sticker (item E) on it, as shown.
8. Post the postal kit using regular royal mail services. There is no need to add any postage to the box

**Thank you very much for taking part in this study!**

### 3. 16S rRNA gene sequencing

For bacterial 16S rRNA gene amplicon sequencing, the variable V3 and V4 regions of the 16S rRNA gene were amplified from genomic DNA using the primers (standard IUPAC nucleotide nomenclature): Forward Primer = 5' TCGTCGGCAGCGTCAGATGTGTATAAGAGACAGCCTACGGGNGGCWGCAGTCGTCGGCAGCGTCAGATGTGTATAAGAGACAGCCTACGGGNGGCWGCAG, Reverse Primer = 5' GTCTCGTGGGCTCGGAGATGTGTATAAGAGACAGGACTACHVGGGTATCTAATCC
The amplicons were then attached with indices and Illumina sequencing adapters using the Nextera XT index kit. The 16S amplicon libraries were pooled and sequenced in an Illumina MiSeq v3 flowcell as 2x300bp paired-end reads.
Quality of raw sequencing data was assessed using FastQC (v0.11.07). Following quality assessment, downstream processing was performed using dada2 (PMID:27214047) which was implemented in OCMS_16S dada2 pipeline (https://github.com/OxfordCMS/OCMS_16S). Given the drop in quality at ~200bp, we decided to proceed with the analysis using the first read in the pair (V3 region). The filterAndTrim function in dada2 was used to truncate the reads to 275bp. Retained primer sequences were removed from reads (17bp) which resulted in final read lengths of 258bp. Error learning, de-replication and sample inference were performed using dada2 with default parameters. Taxonomy was assigned to amplicon sequence variants (ASVs) using the assignTaxonomy function in dada2 and the NCBI RefSeq training data (*https://zenodo.org/record/2541239/files/RefSeq-RDP16S_v2_May2018.fa.gz*).

## 4. Faeces wet and dry weight and weight-adjustment of SCFA concentrations

Both the wet weight and the dry weight of each faecal sample were recorded to generate a ratio of wet to dry weight for all the samples collected at each time point. The wet weight was determined by: (the weight of the tube with sample in fixed solution) – (weight of the tube with fixing solution). The dry weight was determined by lyophilising a section from each of the samples for at least 6hrs. The dry weight of the sample was determined by: (the weight of tube with dry sample) – (weight of the tube).

Both wet weight and dry weights of faecal samples were recorded for purposes of adjusting SCFA concentrations for variability in stool consistency. Wet weight was recorded as the weight of the sample in the collection tube (before subsampling for dry weight calculation) subtracted by the weight of the collection tube. Wet weight adjustment was simply dividing the SCFA concentrations by the wet weight. Dry weight was calculated by lyophilising a fraction of the wet sample, then calculating the dry:wet weight ratio by dividing the weight of the fraction when dried by the weight of the fraction when wet. Dry:wet ratio was then used to extrapolate the dry weight of the sample by multiplying the sample wet weight by the dry:wet weight ratio. Dry weight adjustment was calculated by multiplying the SCFA concentrations by the dry weight.

We found that dietary effects were reflected in the wet weight (and to a lesser extent, the dry weight) of the stool samples. This finding makes intuitive sense as participants have undergone major dietary changes throughout the course of the study, which inevitably impacted the consistency of their stool. While the impact of the TDR on stool consistency is noteworthy, it is a confounding effect on the SCFA. As such, to analyse SCFA levels independent of dietary effects (and focusing our interest on the metabolic (microbial and/or host) responses to the study and given that diet-depended stool weight was more acutely captured by wet weight than dry weight, we decided that adjusting SCFA concentrations by wet weight was favourable over dry-weight adjustment.

Relationship between wet weight and cumulative SCFA levels showed that wet weight of samples is confounded by the dietary conditions.

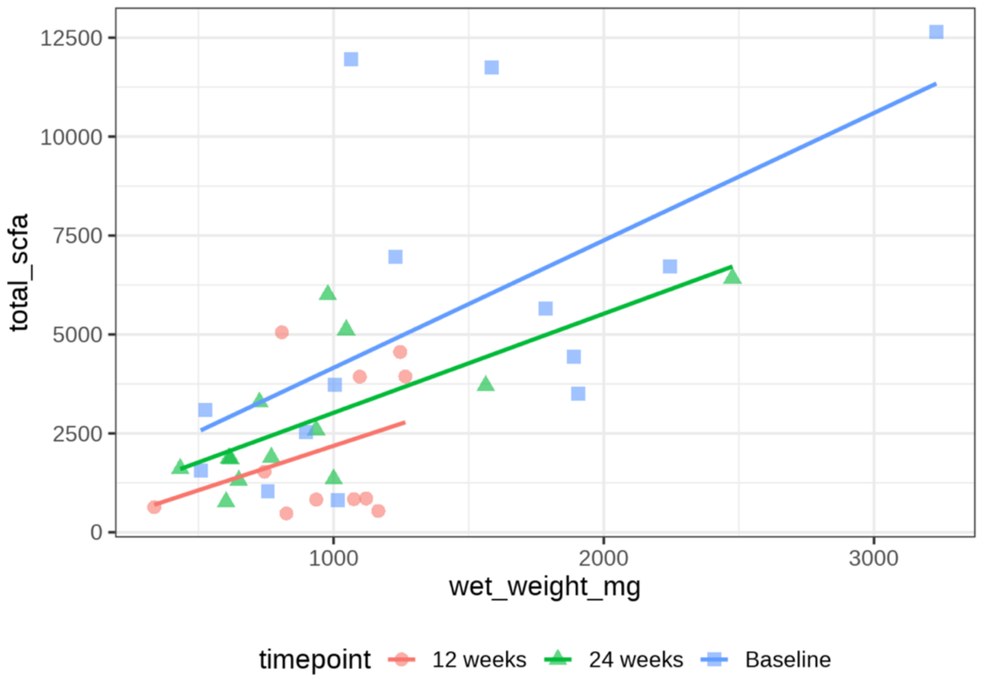


Relationship between cumulative SCFA levels and dry weight shows that diet-dependent effects on stool weight is not as well captured in dry weight as compared to wet weight.

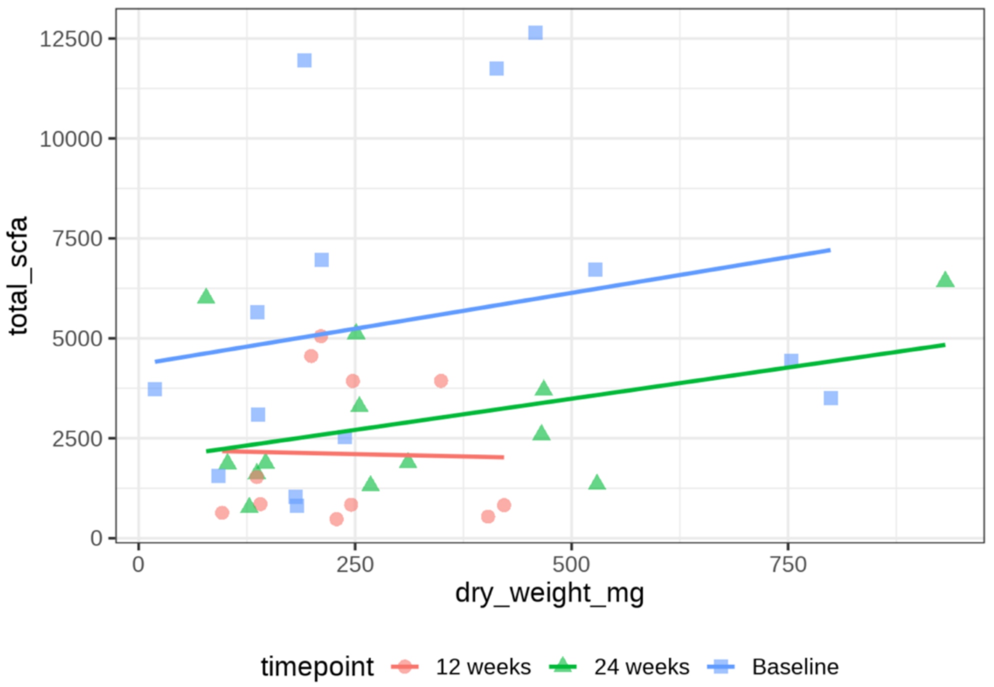

## 5. Ion chromatography-mass spectrometry method

Chromatographic separation was achieved using a partial loop injection (5 µL) of the supernatant to a Dionex ICS-5000+ Capillary HPIC system (Dionex, Sunnyvale, CA, USA) coupled to a Q-Exactive HF hybrid quadrupole-Orbitrap mass spectrometer (Thermo Scientific, San Jose, CA, USA) operating in targeted selected ion monitoring (t-SIM) mode using only MS1 scan and the following m/z [M-H] inclusion list: acetic acid (59.31358); propionic acid (73.0295); isobutyric/ butyric acid (87.04515); isovaleric/ valeric acid (101.0608). Compounds were detected using a Dionex IonPac AS11-HC column (2 × 250 mm2, 4 µm; Dionex, Sunnyvale, CA, USA) column and an aqueous hydroxide ion gradient at a flow rate of 0.25 mL/min with the following steps: 0 min, 0 mM; 1 min, 0 mM; 9 min, 2 mM; 11 min, 100 mM; 14.1 min 0 mM; 16 min 0 mM. A continuously regenerated trap column was used to remove ionic contaminants from the eluent and ion suppression was achieved using a Dionex ERS 500e (Dionex, Sunnyvale, CA, USA) in external water mode with a flow rate of 0.5 ml/min. The mass spectrometer was equipped with a HESI II probe in negative ion mode with source parameters set as follows: sheath gas flow rate, 60; auxiliary gas flow rate, 20; sweep gas flow rate, 0; spray voltage, 3.6 kV; capillary temperature, 300 °C; S-lens RF level, 70 and heater temperature 350 °C. MS1 scan parameters were as follows: resolution, 7 x 105; AGC target, 1 x 106 ions; maximum IT, 175 ms; isolation window, 4.0 m/z.

## 6. Data Handling

This section details on how each data set was handled during analysis.

*Faecal microbiota analysis*

Analysis of the faecal microbiota beta diversity was performed at every taxonomic level. Taxa that did not have at least 5 read counts in any given sample were filtered out prior to analysis, retaining 74-100% of total reads. Where applicable, read counts were normalized by centred log-ratio (CLR) transformation. CLR transformation was performed using the aldex.clr function in the R package ALDEx2, which uses CLR transformation of Monte-Carlo instances drawn from the Dirichlet distribution to estimate per-feature variation within a given sample. Changes in microbiota abundance were calculated as difference between 12-0 weeks and 24-12 weeks, respectively. Where change in microbiota required normalisation, the difference was calculated on the normalised values. Bray Curtis distances were calculated on relative abundances.

*SCFA analysis*

SCFA concentrations were adjusted to the faecal wet weight (see above). Where normalisation was necessary, adjusted concentrations were mean centred and unit-variance scaled. Change in SCFA levels were calculated as the difference between 12-0 weeks and 24-12 weeks, respectively. Where change in SCFA required normalisation, the difference was calculated on the normalised values.

*Markers of intestinal permeability*

Where normalisation was necessary intestinal permeability marker concentrations were mean centred and unit variance scaled. Change in intestinal permeability marker concentrations were calculated as difference between 12-0 weeks and 24-12 weeks, respectively. Where change in intestinal permeability markers required normalisation, the difference was calculated on normalised values.

## 7. Sparse Partial Least Squares Analysis

The relationship between sets of multivariate measures, such as SCFA, liver disease severity markers, and intestinal permeability markers was assessed with sparse partial least squares (sPLS) analysis, a dimension reduction analysis that maximises the co-variance between two multivariate datasets. We conducted sPLS analysis between change in markers of permeability (independent) and change in liver severity (dependent) as well as change in SCFA (independent) and change zonulin (dependent). For those independent variables with variable importance projection (VIP) coefficient >1, we ran mixed effects linear models of change following the principles described in the main paper. We used the Benjamini-Hochberg method to correct for the fact that multiple models were assessed after each sPLS, where applicable. Adjusted p-values<0.05 was deemed statistically significant.

**Figure S1**: LBP for each individual over time. Corresponding MELM in Table S1

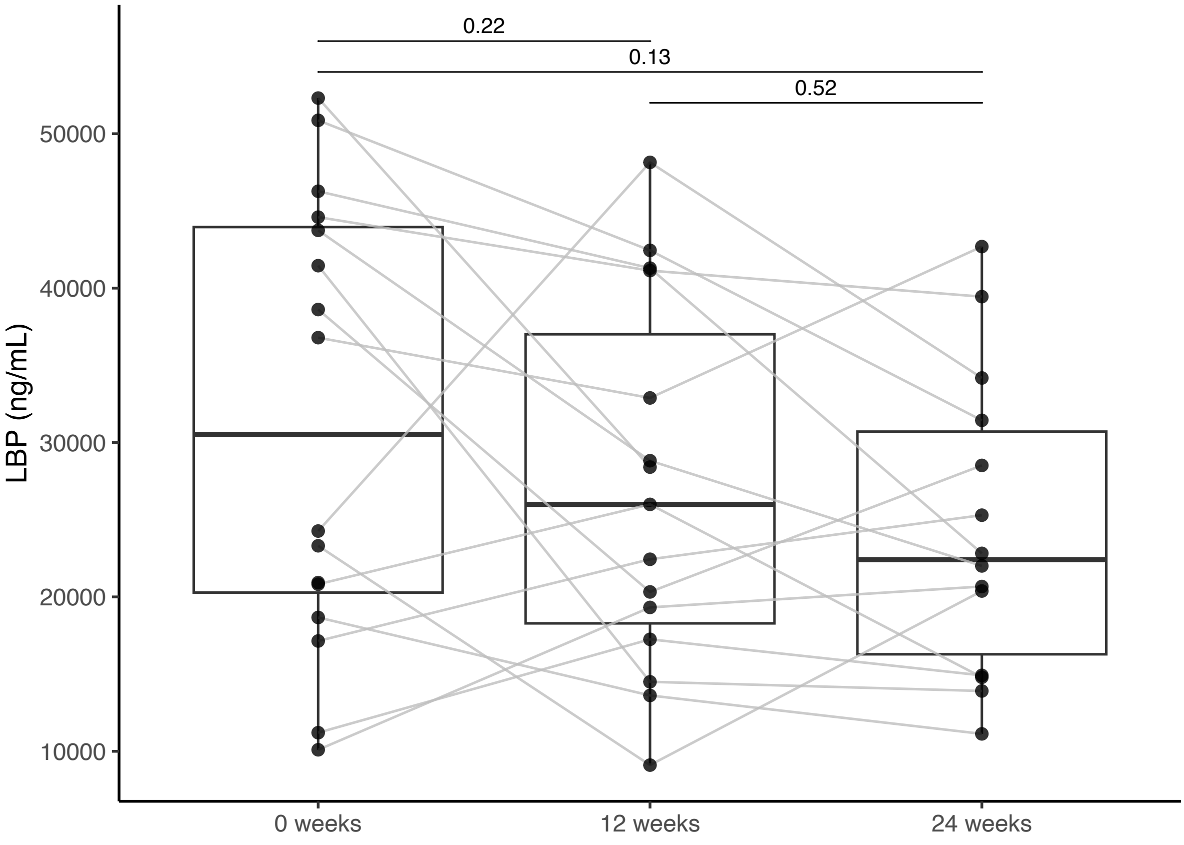

**Figure S2**: Within-individual alpha diversity measured by (A) evenness and (C) richness. (B) Changes in evenness as it relates to changes in weight. (D) Changes in richness as it relates to changes in weight. Within-individual alpha diversity metrics modelled using MELM (Table S7, S8), with difference in LS-means as post-hoc test for pair-wise comparisons using Statterthwaite method.

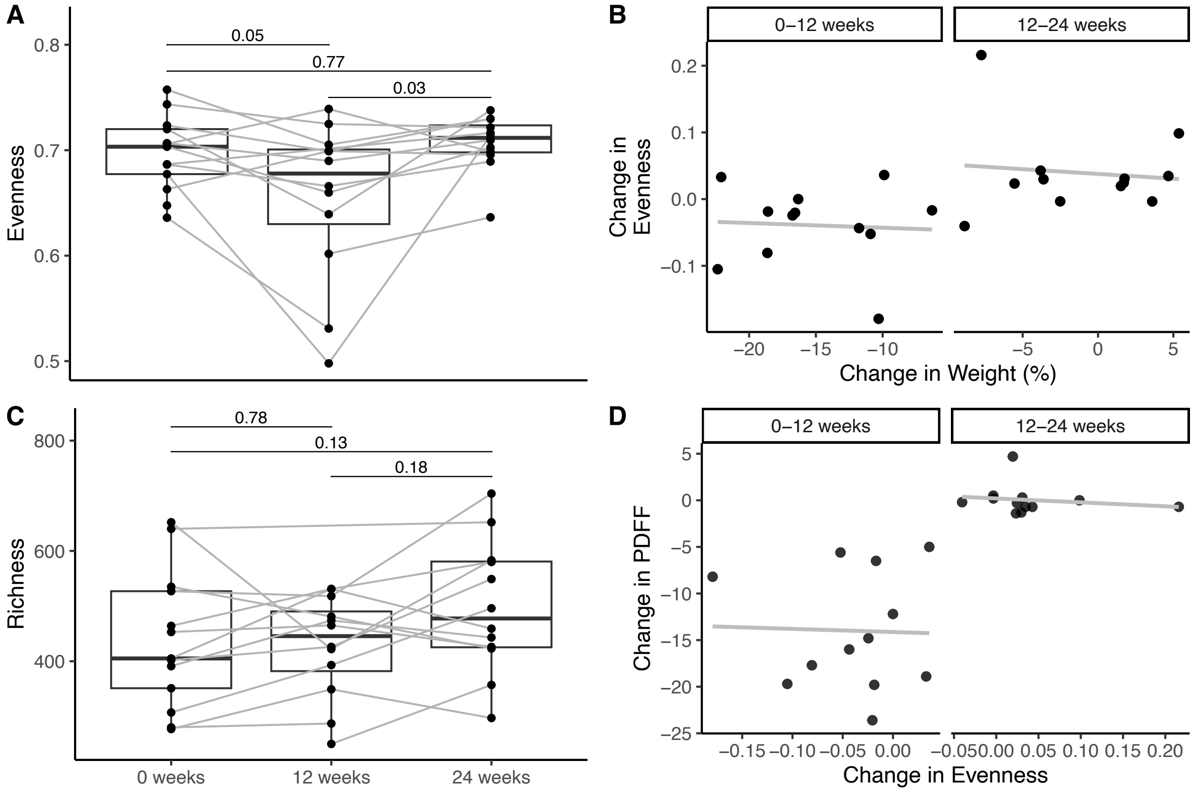

**Figure S3**: Inter-individual beta diversity (Bray-Curtis dissimilarity) at the (A) class, (B) order, (C) family, and (D) genus levels. Inter-individual beta diversity modelled using MELM (Table S10), with difference in LS-means as post-hoc test for pair-wise comparisons using Statterthwaite method.

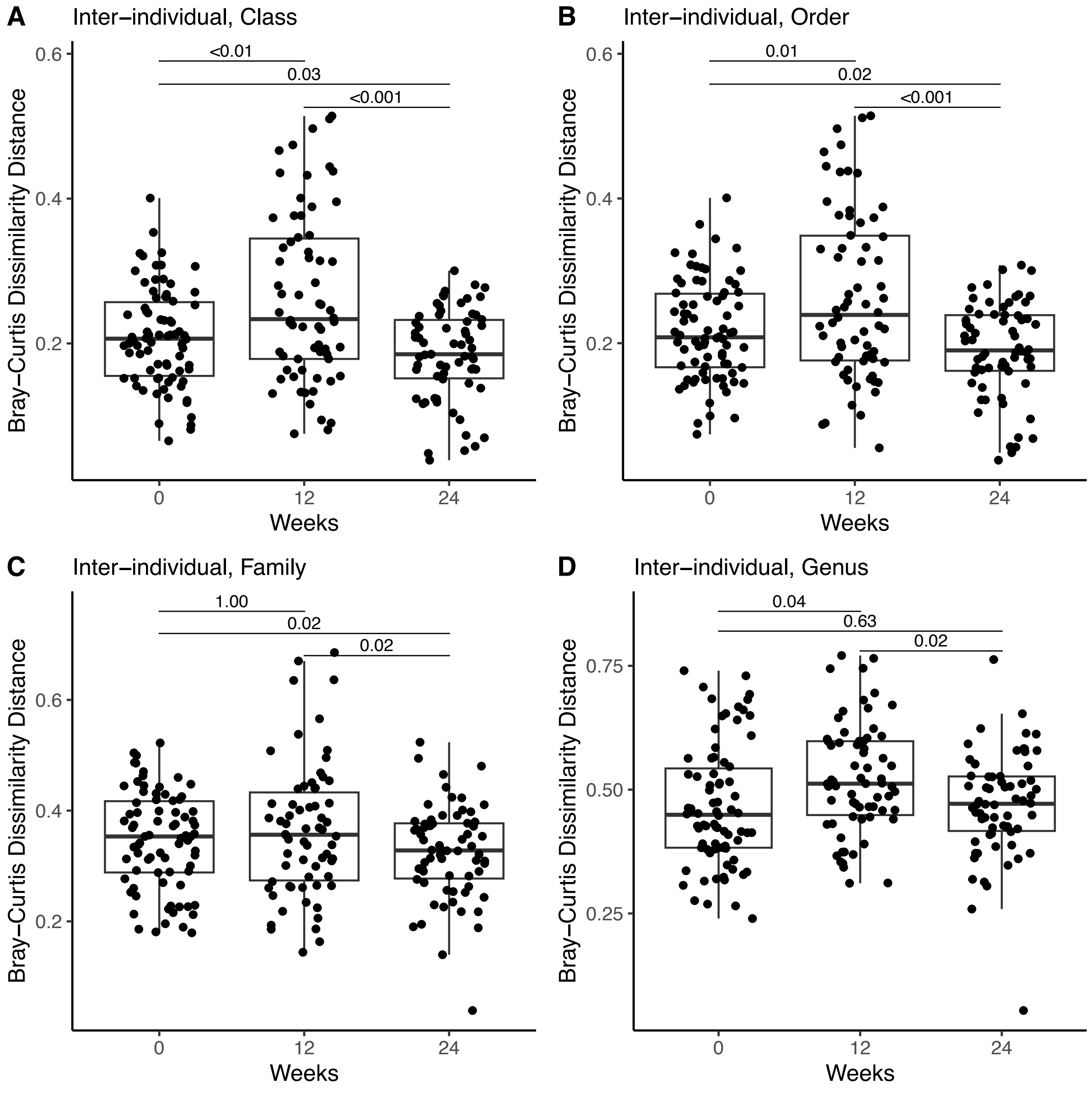

**Figure S4**: Mean relative abundance of the most prevalent taxa at each timepoint at (A) phylum, (B) class, (C) order, (D) family, and (F) genus level. Only taxa in the top 80% abundance are shown.

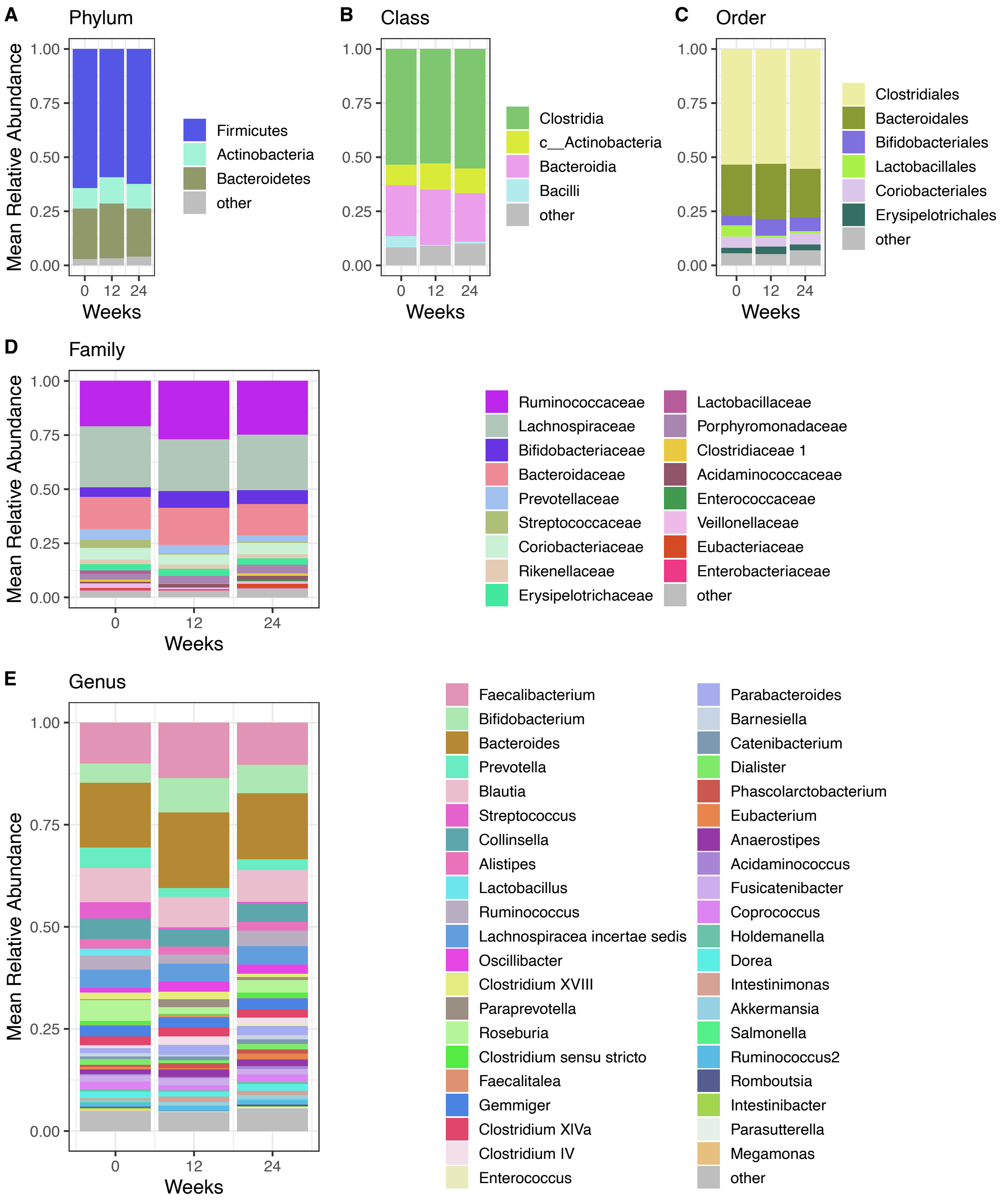

**Figure S5**. Taxa that were differentially abundant when comparing baseline and 12 weeks (restricted) microbiota as detected using mixed effects linear model.

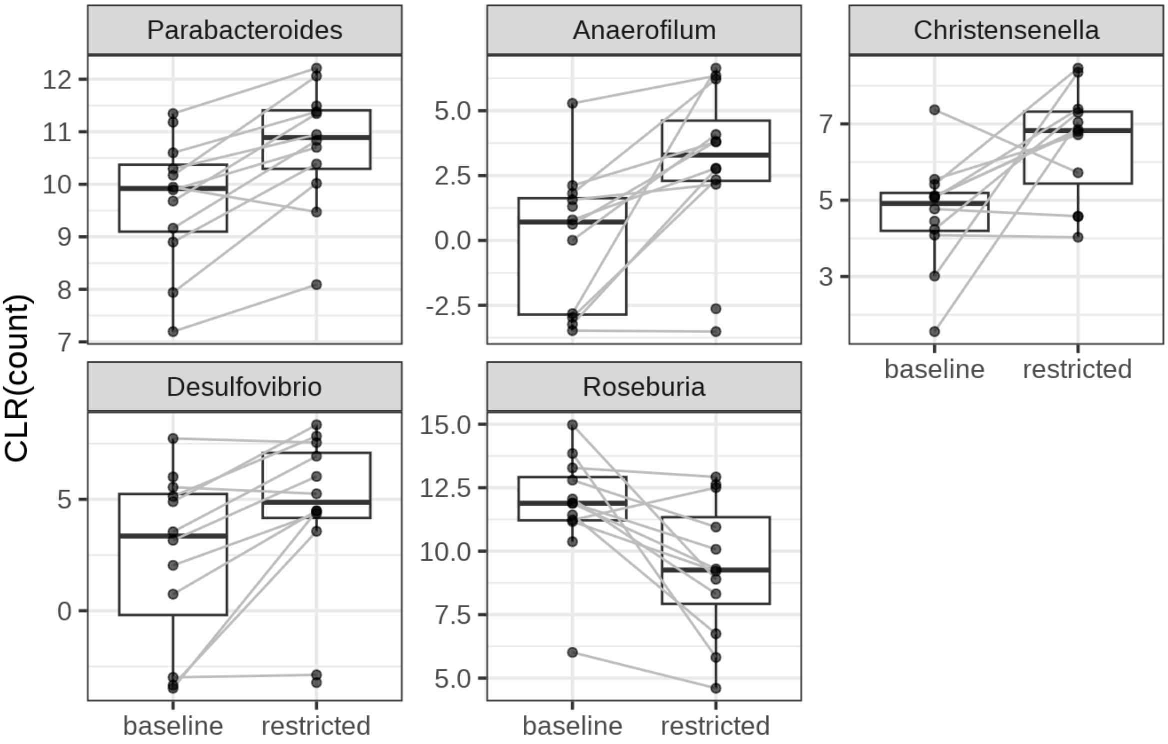

**Table S1**. Mixed effects linear model (MELM) of intestinal permeability over time. Estimates (95% confidence intervals) shown for each model term.

| | | Change from baseline at | |
|---|---|---|---|
| | Intercept | 12 weeks | 24 weeks |
| LBP, ng/mL | 31318.51 (25304.43 to 37332.59) | -4624.91 (-10698.09 to 1507.11) | -6706.57 (-12950.33 to -463.38) |
| Zonulin, ng/mL | 341.27 (279.54 to 403.01) | -184.70 (-248.50 to -122.19) | -152.01 (-217.35 to -88.03) |

**Table S2**. MELM of zonulin against change in weight. Estimates (95% confidence intervals) shown for each term.

| | Intercept | Per 1% change in weight | Timepoint | Interaction, timepoint * change in weight |
|---|---|---|---|---|
| Change in zonulin | 18.58 (-127.43 to 164.60) | 13.17 (3.82 to 22.53) | 17.00 (-135.70 to 169.70) | 1.08 (-12.67 to 14.43) |

**Table S3**. MELM of change in liver fat (proton density fat fraction) against change in zonulin. Estimates (95% confidence intervals) shown for each term.

| | Intercept | Per 1ng/ml change in zonulin | 12 to 24 weeks | Interaction, 12 to 24 weeks * change in zonulin |
|---|---|---|---|---|
| Change in PDFF | -0.43 (-0.73 to -0.13) | 0.60 (0.31 to 0.88) | 1.11 (0.69 to 1.57) | -0.30 (-0.81 to 0.21) |

**Table S4**. SCFA concentrations observed throughout the study, shown as mean (standard deviation).

| Metabolite | Baseline | 12 weeks | 24 weeks |
|---|---|---|---|
| Acetic acid | 2.57 (1.80) | 1.44 (1.21) | 1.95 (1.09) |
| Propionic acid | 0.70 (0.54) | 0.32 (0.26) | 0.44 (0.22) |
| Butyric acid | 0.52 (0.43) | 0.23 (0.18) | 0.48 (0.24) |
| Isovaleric acid | 0.09 (0.15) | 0.04 (0.03) | 0.07 (0.04) |
| Valeric acid | 0.08 (0.06) | 0.04 (0.03) | 0.08 (0.03) |
| Isobutyric acid | 0.06 (0.05) | 0.11 (0.22) | 0.07 (0.03) |

**Table S5**. MELM of cumulative SCFA, SCFA dispersion, and individual SCFAs, over time. Estimates and 95% confidence intervals shown for each model term.

| | Intercept | 12 weeks | 24 weeks |
|---|---|---|---|
| cumulative SCFA | 4.02<br>(2.91 to 5.12) | -1.78<br>(-3.15 to -0.42) | -0.77<br>(-2.09 to 0.50) |
| SCFA dispersion | 2.68<br>(2.06 to 3.30) | -1.21<br>(-2.05 to -0.35) | -1.14<br>(-1.99 to -0.35) |
| Acetic acid | 2.57<br>(1.83 to 3.32) | -1.10<br>(-2.05 to -0.15) | -0.53<br>(-1.45 to 0.36) |
| Butyric acid | 0.52<br>(0.35 to 0.68) | -0.28<br>(-0.47 to -0.08) | -0.02<br>(-0.20 to 0.16) |
| Isobutyric acid | 0.06<br>(-0.00 to 0.12) | 0.05<br>(-0.04 to 0.15) | 0.02<br>(-0.08 to 0.10) |
| Isovaleric acid | 0.09<br>(0.04 to 0.14) | -0.05<br>(-0.12 to 0.03) | -0.02<br>(-0.09 to 0.05) |
| Propionic acid | 0.70<br>(0.50 to 0.90) | -0.38<br>(-0.63 to -0.13) | -0.24<br>(-0.48 to -0.01) |
| Valeric acid | 0.08<br>(0.05 to 0.10) | -0.03<br>(-0.07 to -0.00) | 0.00<br>(-0.03 to 0.03) |

**Table S6**. MELM of changes in individual SCFAs against changes in weight. Estimates (95% CI) shown for each model term.

| | Intercept | Per 1% change in weight | Timepoint | Interaction, timepoint * change in weight |
|---|---|---|---|---|
| Change in acetic acid | 1.23<br>(-2.37 to 4.83) | 0.14<br>(-0.07 to 0.36) | -0.59<br>(-4.30 to 3.08) | -0.08<br>(-0.37 to 0.22) |
| Change in propionic acid | 0.60<br>(-0.30 to 1.50) | 0.06<br>(0.01 to 0.11) | -0.44<br>(-1.36 to 0.48) | -0.04<br>(-0.12 to 0.03) |
| Change in isobutyric acid | -0.01<br>(-0.25 to 0.23) | -0.00<br>(-0.02 to 0.01) | 0.10<br>(-0.09 to 0.32) | 0.01<br>(-0.01 to 0.03) |
| Change in butyric acid | 0.38<br>(-0.30 to 1.05) | 0.04<br>(0.00 to 0.08) | -0.17<br>(-0.86 to 0.52) | -0.03<br>(-0.09 to 0.02) |
| Change in isovaleric acid | -0.33<br>(-0.63 to -0.03) | -0.02<br>(-0.04 to 0.00) | 0.36<br>(0.05 to 0.67) | 0.02<br>(-0.01 to 0.04) |
| Change in valeric acid | 0.03<br>(-0.08 to 0.15) | 0.00<br>(-0.00 to 0.01) | -0.00<br>(-0.12 to 0.11) | -0.00<br>(-0.01 to 0.01) |

**Table S7**. MELM of alpha diversity metrics over time. Estimates (95% confidence interval) shown for each term.

| | | Change from baseline at | |
|---|---|---|---|
| | Intercept | 12 weeks | 24 weeks |
| Shannon's D | 68.76<br>(55.69 to 81.61) | -9.31<br>(-21.60 to 2.83) | 10.11<br>(-2.00 to 22.45) |
| Richness | 427.66<br>(364.25 to 490.17) | 8.02<br>(-47.56 to 62.25) | 55.67<br>(1.19 to 110.71) |
| Evenness | 0.69<br>(0.67 to 0.72) | -0.04<br>(-0.07 to -0.01) | 0.01<br>(-0.02 to 0.04) |

**Table S8**. MELM of changes in evenness and Shannon's D against changes in weight. Estimates (95% confidence intervals) shown for every model term.

| | Intercept | Per 1% change in weight | Timepoint | Interaction, timepoint * change in weight |
|---|---|---|---|---|
| Change in Evenness | -0.05<br>(-0.16 to 0.06) | -0.00<br>(-0.01 to 0.01) | 0.09<br>(-0.03 to 0.21) | -0.00<br>(-0.01 to 0.01) |
| Change in Shannon's D | -23.89<br>(-66.36 to 18.59) | -1.00<br>(-3.69 to 1.68) | 41.58<br>(-3.00 to 86.17) | 1.09<br>(-2.81 to 4.99) |

**Table S9**. MELM of changes in liver severity markers against changes in evenness

| | Intercept | Per 1-point change in evenness | Timepoint | Interaction, timepoint * change in evenness |
|---|---|---|---|---|
| Change in PDFF | -14.15<br>(-17.31 to -10.99) | -3.13<br>(-48.36 to 42.39) | 14.40<br>(10.31 to 18.40) | -5.38<br>(-80.28 to 62.95) |

**Table S10**. MELM of inter-individual beta diversity over time. Estimates (95% confidence interval) shown for each model term.

| | | Change from baseline at | |
|---|---|---|---|
| | Intercept | 12 weeks | 24 weeks |
| Phylum level | 0.14 | 0.08 | -0.02 |
| Bray-Curtis distance | (0.11 to 0.17) | (0.06 to 0.10) | (-0.04 to 0.01) |
| Class level | 0.21 | 0.04 | -0.03 |
| Bray-Curtis distance | (0.19 to 0.24) | (0.02 to 0.07) | (-0.06 to -0.00) |
| Order level | 0.22 | 0.04 | -0.03 |
| Bray-Curtis distance | (0.19 to 0.25) | (0.01 to 0.06) | (-0.06 to -0.01) |
| Family level | 0.35 | -0.00 | -0.04 |
| Bray-Curtis distance | (0.31 to 0.38) | (-0.03 to 0.03) | (-0.06 to -0.01) |
| Genus level | 0.47 | 0.04 | -0.01 |
| Bray-Curtis distance | (0.43 to 0.50) | (0.00 to 0.07) | (-0.04 to 0.03) |

## References

[1] Triay Bagur A, Hutton C, Irving B, Gyngell ML, Robson MD, Brady M. Magnitude-intrinsic water-fat ambiguity can be resolved with multipeak fat modeling and a multipoint search method. Magn Reson Med 2019;82:460-475.
[2] Piechnik SK, Ferreira VM, Dall'Armellina E, Cochlin LE, Greiser A, Neubauer S, Robson MD. Shortened Modified Look-Locker Inversion recovery (ShMOLLI) for clinical myocardial T1-mapping at 1.5 and 3 T within a 9 heartbeat breathhold. J Cardiovasc Magn Reson 2010;12:69.
[3] Tunnicliffe EM, Banerjee R, Pavlides M, Neubauer S, Robson MD. A model for hepatic fibrosis: the competing effects of cell loss and iron on shortened modified Look-Locker inversion recovery T1 (shMOLLI-T1 ) in the liver. J Magn Reson Imaging 2017;45:450-462.
[4] Mojtahed A, Kelly CJ, Herlihy AH, Kin S, Wilman HR, McKay A, et al. Reference range of liver corrected T1 values in a population at low risk for fatty liver disease-a UK Biobank sub-study, with an appendix of interesting cases. Abdom Radiol (NY) 2019;44:72-84.
[5] QIBA. Magnetic Resonance Elastography of the Liver. 2018 [cited 11 July 2022]; Available from: https://qibawiki.rsna.org/images/a/a5/MRE-QIBAProfile-2018-05-02-CONSENSUS.pdf
